# Endothelial Dysfunction and Passive Changes in the Aorta and Coronary Arteries of Diabetic db/db Mice

**DOI:** 10.3389/fphys.2020.00667

**Published:** 2020-06-23

**Authors:** Lilliana Beck, Junjing Su, Simon Comerma-Steffensen, Estéfano Pinilla, Rune Carlsson, Raquel Hernanz, Majid Sheykhzade, Carl Christian Danielsen, Ulf Simonsen

**Affiliations:** ^1^Department of Biomedicine, Pulmonary and Cardiovascular Pharmacology, Faculty of Health Aarhus University, Aarhus, Denmark; ^2^Department of Biomedical Sciences/Animal Physiology, Veterinary Faculty, Central University of Venezuela, Maracay, Venezuela; ^3^Departamento de Ciencias Básicas de la Salud, Universidad Rey Juan Carlos, Alcorcón, Spain; ^4^Department of Drug Design and Pharmacology, Faculty of Health and Medical Sciences, University of Copenhagen, Copenhagen, Denmark

**Keywords:** acetylcholine, endothelium, aorta, coronary arteries, stiffness, viscoelasticity

## Abstract

Endothelial cell dysfunction and vessel stiffening are associated with a worsened prognosis in diabetic patients with cardiovascular diseases. The present study hypothesized that sex impacts endothelial dysfunction and structural changes in arteries from diabetic mice. In diabetic (db/db) and normoglycaemic (db/db+) mice, the mechanical properties were investigated in pressurized isolated left anterior descending coronary arteries and aorta segments that were subjected to tensile testing. Functional studies were performed on wire-mounted vascular segments. The male and female db/db mice were hyperglycaemic and had markedly increased body weight. In isolated aorta segments without the contribution of smooth muscle cells, load to rupture, viscoelasticity, and collagen content were decreased suggesting larger distensibility of the arterial wall in both male and female db/db mice. In male db/db aorta segments with smooth muscle cell contribution, lumen diameter was smaller and the passive stretch-tension curve was leftward-shifted, while they were unaltered in female db/db aorta segments versus control db/db+ mice. In contrast to female db/db mice, coronary arteries from male db/db mice had altered stress-strain relationships and increased distensibility. Transthoracic echocardiography revealed a dilated left ventricle with unaltered cardiac output, while aortic flow velocity was decreased in male db/db mice. Impairment of acetylcholine relaxation was aggravated in aorta from female db/db compared to control and male db/db mice, while impairment of sodium nitroprusside relaxations was only observed in aorta from male db/db mice. The remodeling in the coronary arteries and aorta suggests an adaptation of the arterial wall to the reduced flow velocity with sex-specific differences in the passive properties of aorta and coronary arteries. The findings of less distensible arteries and more pronounced endothelial dysfunction in female compared to male diabetic mice may have implications for the observed higher incidence of macrovascular complications in diabetic women.

## Introduction

Patients with diabetes have a greater risk of atherosclerosis and cardiovascular complications than non-diabetics (Rydén et al., 2013). Genetic disposition, hyperglycaemia, production of advanced glycation end products (AGEs), hyperlipidemia, insulin resistance, and hypertension have been reported to explain the accelerated diabetic macroangiopathy and progression to cardiovascular disease. Diabetic macroangiopathy is characterized by increased vessel stiffness, thickness, and endothelial dysfunction (Sasaki et al., 2008; Prenner and Chirinos, 2015).

Nitric oxide (NO) derived from the endothelium has a variety of functions including regulation of vascular tone, inhibition of platelet aggregation and adhesion of inflammatory leukocytes to the endothelial cells, and therefore endothelial dysfunction is thought to be of importance for the development of atherosclerosis and nephropathy in diabetes (Paneni et al., 2013). In animal models and humans with types 1 and 2 diabetes mellitus, endothelium-dependent vasodilatation is impaired in both large and small arteries (Caballero et al., 1999; Pannirselvam et al., 2002; Gao et al., 2007, 2008; Zhang et al., 2008; Bagi et al., 2015; Chen et al., 2015; Lee et al., 2017; Katare et al., 2018). Acute hyperglycaemia is also associated with impaired endothelium-dependent vasodilatation and decreased NO bioavailability (Brodsky et al., 2017), and inactivation of NO by oxygen-derived free radical species is thought to be an important mechanism in this condition (Pannirselvam et al., 2002; Gao et al., 2007; Paneni et al., 2013). Hyperglycaemia and oxidative stress lead to increased production of AGEs and a further reduction in NO production (Gao et al., 2008; Farmer and Kennedy, 2009), but the consequences of the endothelial dysfunction on the altered structure and vascular stiffening are unclear.

In addition to endothelial dysfunction, diabetic macroangiopathy is characterized by increased extracellular matrix deposition around the smooth muscle cells and vessel stiffing which by itself is predictive of cardiovascular disease progression (Hansen et al., 2006; Vlachopoulos et al., 2010). In type 2 diabetic db/db mice, aorta, mesenteric arteries, and septal coronary arteries seem to respond in a tissue-specific manner (Katz et al., 2011), as vessel stiffness seems to be increased in aorta and mesenteric arteries, while septal coronary arteries respond with decreased intraluminal diameter and decreased stiffness (Katz et al., 2011; Husarek et al., 2015). Comparing intact and decellularized aorta and coronary microvessels from db/db mice which allow investigation of the extracellular matrix also suggested that the microvessels are more influenced by the adverse consequences of type 2 diabetes at earlier stages of disease progression than conduit arteries (Anghelescu et al., 2015).

Changes in endothelial function and stiffness have been reported independently for different vascular beds (Bagi et al., 2005, 2015; Zhang et al., 2008; Katz et al., 2011; Anghelescu et al., 2015; Husarek et al., 2015). Although the physiological relevance of local aldosterone production remains unclear, recent studies have suggested that vascular smooth muscle, as well as perivascular adipocyte production of aldosterone, may contribute to vascular dysfunction in obese and diabetic mice (Briones et al., 2012; Matsuzawa et al., 2013). These studies provide support to the assumption that the development of endothelial dysfunction followed by altered stiffness and vascular structure is not necessarily global, but it may be specific for each vascular bed in diabetes.

Sex and obesity also impact the development of cardiovascular complications in diabetes. Obesity is a major risk factor of type 2 diabetes in both sexes, and while men are more obese at young age, women above 45 years are often overweight or tend to be more obese than men (Kautzky-Willer et al., 2016). Moreover, diabetic men appear at a higher risk for diabetic microvascular complications, while the consequences of macrovascular complications may be greater in women (Maric-Bilkan, 2017). Compared to diabetic men, diabetes in women is associated with greater increases of cardiovascular risk, myocardial infarction, and stroke mortality. This is not the case with non-diabetic subjects (Peters et al., 2014). However, with few exceptions, most of the studies in animal models have been conducted in males (Docherty et al., 2019).

The present study hypothesized that sex impacts endothelial dysfunction and structural changes in arteries from diabetic mice. Diabetic (db/db) mice develop obesity, insulin resistance, hyperglycaemia, and hyperlipidemia similar to that seen in human type 2 diabetes due to a spontaneous mutation in the leptin receptor gene (Hummel et al., 1966). To address the hypothesis, we investigated the structural changes as well as stiffness and endothelial function in aorta from female and male control and diabetic mice. Given that most studies in coronary arteries from db/db mice studied septal arteries (Katz et al., 2011; Husarek et al., 2015; McCallinhart et al., 2018) or coronary microvessels (Zhang et al., 2008; Lee et al., 2017), we investigated whether the same structural changes take place in the left anterior descending (LAD) coronary arteries from male and female diabetic mice. Previous studies have found impaired left ventricle function in diabetic db/db mice (Plante et al., 2014; Baumgardt et al., 2016; Moon et al., 2016; Xu et al., 2016) and even suggested that cardiac dysfunction may precede vascular dysfunction in diabetic mice (Katare et al., 2018). Therefore, we asked also whether vascular changes can be explained by changes in cardiac function and aortic flow, and therefore transthoracic echocardiography and blood pressure measurements was performed in a subset of male control and diabetic mice.

## Materials and Methods

### Animals and Preparation of Samples

Male and female 8-week-old db/db mice (C57BLKS/J-lepr^*db*^/lepr^*db*^) and age- and sex-matched db/db+ littermate controls (C57BLKS/J-lepr^*db*^/lepr^*db+*^) were purchased from Taconic Europe (Ry, Denmark). The mice were housed in the animal facility in 365 × 207 × 140 mm cages with standard wood bedding and space for three to four mice, and they had free access to water and laboratory diet (Brogaarden, Middelfart, Denmark). The mice had a 12-hour light/dark cycle, and to avoid influence of torpor (Swoap and Gutilla, 2009; Ayala et al., 2010), the mice were allocated to experimentation early in the light period. Glucose was monitored weekly in non-fasting animals by use of arterial tail blood. Blood glucose was measured twice each time with a Contour Blood Glucose Monitoring System (Bayer, Copenhagen, Denmark). The mice were killed by decapitation followed by exsanguination for the mechanical and the functional studies. All animal care and experimental protocols in this study were conducted under the supervision of a veterinarian and in accordance with the Danish legislation of animal use for scientific procedures as described in the “Animal Testing Act” (Consolidation Act No. 726 of 9 September 1993 as amended by Act No. 1081 of 20 December 1995) and approved by the Danish Animal Experiments Inspectorate (permission 2014-15-2934-01059). The Danish Animal Testing Act fully and extensively covers the requirements included in the Guide for the Care and Use of Laboratory Animals as adopted and promulgated by the United States National Health Institute, and the animal studies are reported in compliance with the ARRIVE guidelines (Kilkenny et al., 2010; McGrath and Lilley, 2015). The mice were examined at 16 weeks of age.

### Passive Mechanical Studies in Aorta Segments

Mechanical testing was performed on aorta segments from 10 db/db and 8 db/db+ male mice to 12 db/db and 14 db/db + female mice. Three to five ring specimens with a height of 1 mm were cut from the frozen descending thoracic aorta and placed in a 50 mM Tris/HCl buffer (pH 7.4) at room temperature. Side branches were avoided in the cutting process. The cross-sectional area of the specimens was measured (Supplementary Figure S1), and afterward, the specimens were frozen at −20°C until analysis at which time specimens were thawed at room temperature.

The in-house built setup for mechanical testing has previously been described (Van Soldt et al., 2015; Filogonio et al., 2018). Briefly, the specimen was mounted around two parallel, orthogonally bent spring steel hooks with a diameter of 0.35 mm and submerged in the Tris/HCl buffer at room temperature. One hook was connected to a load cell of 3 N capacity (Kistler-Morse DSC-6 transducer, DMT, Aarhus, Denmark) and the other hook was moved by a step motor (DM224i API Motion, Amherst, NY, United States) at 0.1667 mm/s. Deformation was obtained from the step motor drive and load cell readings were acquired by a data acquisition unit [Model 34970A, Hewlett Packard (Keysight), Palo Alto, CA, United States]. Each specimen was first subjected to five loading cycles to 35 mN and then to a rupture test. The ruptured specimens were collected for collagen analysis.

From the fifth hysteresis cycle, the area between the loading and de-loading load-deformation curves (the energy of the hysteresis loop) was calculated in percent of the loading area. This percentage expresses the relative importance of viscosity, whereas the energy recovered to the total energy input indicates a higher elastic efficiency (Shadwick, 1992). The initial luminal perimeter, l_0_, was obtained from the rupture load-deformation curve where the load increased to above a minimal value (0.1 mN) during deformation. Maximal stiffness was calculated as the maximal slope of the load-strain curve. Stiffness and strain corresponding to pressures, P, of 100 and 120 mm Hg were estimated from the load-strain curve by finding the combination of load (F) and strain fulfilling the Laplace equation (see Van Soldt et al., 2015) for P (*P* = *F*/(*r* × *h*), where *r* = l/2π and *h* = 1 mm and l is height and luminal perimeter, respectively, of ring specimen). Maximal stress and maximal modulus and maximal mechanical quality of collagen were estimated by normalization of maximal load and maximal stiffness by cross-sectional area and unit collagen [UC, mg collagen/mm initial luminal perimeter (l_0_)], respectively.

### Functional Studies of Aorta Segments

Freezing and thawing of the samples do not allow to take into account the endothelial function and vessel tone determined by the vascular smooth muscle. Therefore, in a different set of mice (11 male db/db+ and 11 db/db, and 12 female db/db+ and 10 db/db mice) the descending thoracic aorta was placed in physiological salt solution (PSS) immediately after euthanasia and ring segments of 2 mm were dissected and mounted on two 100 μm steel wires in microvascular myographs (Danish Myo Technology, Aarhus, Denmark) for isometric tension recordings. PSS was of the following composition (mmol/L): NaCl 119, KCl 4.7, KH_2_PO_4_ 1.18, MgSO_4_ 1.17, NaHCO_3_ 25, CaCl_2_ 1.6, EDTA 0.026 and glucose 5.5. To avoid acute hyperglycaemic effects on endothelial function (Bangshaab et al., 2019), which may confound the relation of endothelial to structural function, experiments on arteries from both control db/db+ and diabetic db/db male and female mice were conducted with the same glucose concentration. The organ baths were heated to 37°C and equilibrated with 5% CO_2_ to maintain the desired pH of 7.4. The segments were allowed to equilibrate for 10 min. To determine the optimal passive to an active relationship in the aorta, the segments were incrementally stretched by increasing the internal circumference (IC) successively with 100 μm and then stimulated with potassium-rich PSS (KPSS, 60 mM) each time.

For the initial viability test, we tested the endothelial cell function; aorta segments were contracted with phenylephrine (10^–7^ M), and after reaching a stable plateau, concentration-response curves were constructed for acetylcholine (10^–9^ to 10^–5^ M) and the NO donor sodium nitroprusside (SNP, 10^–10^ to 10^–5^ M).

### Determination of Collagen and Elastin Content in Aorta

Collagen content was determined for each of the aorta ring segments from male and female mice that were subjected to passive mechanical testing. Besides, the fractional aorta collagen and elastin content were determined relative to dry defatted weight. Thus, four pooled samples of aorta pieces from 10 db/db to 9 db/db+ male mice were defatted with acetone and freeze-dried. Elastin content (dry weight) was determined after extraction of all other tissue components (Lansing et al., 1952) and collagen content was determined from hydroxyproline in the extract. Hydroxyproline content was determined by colorimetric determination after a modified Woessner method (Danielsen and Andreassen, 1988). Collagen was calculated as 7.46 × hydroxyproline content (Neuman and Logan, 1950).

### Passive Properties of Coronary Arteries

To determine the passive properties of the left anterior descending coronary artery (LAD) from 6 male db/db+ and 6 db/db mice and from female 6 db/db+ and 6 db/db mice, arterial segments (lengths of 1.7 ± 0.5 mm) were mounted in a 110P pressure myograph system (Danish Myo Technology, Aarhus, Denmark) in a calcium-free PSS. During the dissection of the LAD, as much myocardium as possible was carefully removed to enable optimal measurements of the internal and external diameters. Furthermore, any visible side-branches were ligated. Once mounted on the myograph, the vessels were unbuckled by the adjustment of the cannulas at 100 mmHg intraluminal pressure and the inner and outer diameters were recorded. It was possible to calculate the structural and mechanical parameters from the internal (Di) and external (De) vessel diameters measured under passive conditions at intraluminal pressures ranging from 10 to 140 mmHg. The passive properties of the coronary arteries mounted in the pressure myograph were calculated as previously described (Supplementary Table S1; Hernanz et al., 2012; Schjørring et al., 2015).

### Functional Studies in Coronary Arteries

Functional studies in coronary arteries were performed on segments from 6 db/db+ and 6 db/db male mice. Due to technical difficulties, the groups were not of equal size and did not include coronary arteries from female mice. Coronary small arteries with a length of approximately 2 mm were mounted on two 25 μm wires in microvascular myographs (Danish Myo Technology, Aarhus, Denmark) for isometric tension recordings as described above for the aorta segments. The segments were stretched to their optimal diameter, which corresponded to an internal circumference of 90% of that achieved when the vessels were exposed to a passive tension yielding a transmural pressure of 100 mmHg in coronary arteries (Simonsen et al., 1999). The viability of the segments was confirmed by their ability to contract to first potassium-rich PSS (KPSS, 60 mM) and subsequently to the thromboxane analog U46619 (10^–7^ M). In U46619-contracted segments, after reaching a stable plateau, concentration-response curves were constructed for acetylcholine (10^9^–10^–5^ M) and SNP (10^–10^–10^–5^ M).

The following drugs were used: Acetylcholine, phenylephrine hydrochloride (PE), SNP (sodium nitroprusside), and U46619 (9α-epoxymethanoprostaglandin F_2α_) were from Sigma (St Louis, MO, United States). Unless otherwise stated, the substances were dissolved in distilled water. The vehicle concentration in the bath was low, and parallel control curves were run to examine whether the vehicle affected vascular contractility.

### Transthoracic Echocardiography

Transthoracic echocardiography was performed on 7 db/db and 7 db/db+ male mice. Anesthesia was induced with 7% sevoflurane mixed in 100% oxygen with the mouse placed in a clear Perspex induction chamber and maintained with 3–4% sevoflurane through a nose mask. Echocardiography (Vevo2100, Visual Sonics, Toronto, Canada) was performed using a linear array probe (MS 550D, 22–55 MHz) on the spontaneously breathing mouse that was placed in the left lateral decubitus position on a heating pad adjusted to 37°C. The animal’s electrocardiography (ECG) signal was captured through copper electrodes on the heating pad. Images of the left ventricle (LV, B-mode, and M-mode) were acquired from the left parasternal short-axis view at mid-papillary level and images of the aorta and pulmonary artery (B-mode and pulse wave Doppler mode) were acquired from the right and left parasternal long-axis views, respectively, (Pistner et al., 2010). Following echocardiography, the mice underwent invasive mean arterial measurement as described below.

Image analysis was performed using the in-built software. All measurements were taken as an average of three consecutive cardiac cycles. The duration of P-wave, the duration of the PR segment (defined as the segment between the end of the P-wave and the beginning of R-wave) and QT-interval were measured (Supplementary Figure S4). PR-interval was calculated as the sum of the durations of P-wave and PR-segment, and the QT interval was corrected for heart rate yielding QT_C_. LV dimensions were measured, and LV function was evaluated (Gao et al., 2011) using the equations in Table 1. Stiffness of the ascending aorta was assessed by its circumferential strain (Trachet et al., 2015), and cardiac output was estimated as the average of the aortic and pulmonary flow (Tournoux et al., 2011; Slama et al., 2015).

**TABLE 1:** Calculation of ventricular function and aortic circumferential strain.

LV FS	$\frac{LVID_{d}-LVID_{s}}{LVID_{d}}\cdot 100\%$
LV EF	$\frac{LVID_{d}^{3}-LVID_{s}^{3}}{LVID_{d}^{3}}\cdot 100\%$
LV FAC	$\frac{Area_{d}-Area_{s}}{Area_{d}}\cdot 100\%$
LVPW thickening	$\frac{LVPW_{s}-LVPW_{d}}{LVPW_{d}}\cdot 100\%$
Aorta circumferential strain	$\frac{1}{2}\cdot \left({\left(\frac{ID_{AO,s}}{ID_{AO,d}}\right)}^{2}-1\right)\cdot 100\%$
Aortic flow	π⋅(*ID*_*AO*_/2)^2^⋅*VTI*_*AO*_⋅*HR*
Pulmonary flow	π⋅(*ID*_*PA*_/2)^2^⋅*VTI*_*PA*_⋅*HR*

*AO: aorta, d: diastole, EF: ejection fraction, FAC: fractional area change, FS: fractional shortening, HR: heart rate, ID: inner diameter, LV: left ventricle, LVID: left ventricular inner diameter, LVPW: left ventricular posterior wall, PA: pulmonary artery s: systole, VTI: velocity time integral.*

### Mean Arterial Pressure Measurement

For *in vivo* arterial pressure measurements, male mice were anesthetized with intraperitoneal administration of pentobarbital (50 mg/kg). The pain was assessed regularly during surgery and pressure measurements by pressing a needle against the paw. In the case of a reaction, additional anesthesia (30 mg/kg pentobarbital) was administered. The carotid artery was isolated following a mid-neck-incision, and a water-filled disposable catheter (MLT0699, ADInstruments, United Kingdom) was inserted. The catheter was fixed afterward by a couple of sutures of 6-0 silk (Johnson & Johnson, Belgium) connected to a four channels PowerLab system (ML485, ADInstruments, United Kingdom), which was connected to a computer that runs LabChart v7 (ADInstruments, United Kingdom). Mean arterial blood pressure (MAP) was measured within a 5 min of stable pulsatile wave pulse. The mice were euthanized by injecting a high dose of pentobarbital (100 mg/kg), and tissue was isolated for *in vitro* protocols.

### Data Evaluation and Statistical Analysis

Data are presented as means ± standard error of the mean (SEM) or median (interquartile range). Statistical analyses were performed using Stata 13 (StataCorp, College Station, TX, United States) or GraphPad Prism 7.02 (GraphPad Software Inc., California, United States). Differences between the db/db+ and db/db mice were compared by the unpaired Student’s *t*-test, Welch’s *t*-test for unequal variances, or Mann–Whitney test (non-parametric test), where appropriate. Concentration-response curves were constructed using a non-linear curve-fit model, and the half-maximal relaxation (EC_50_) was determined:

$$y=bottom+\frac{\left(top-bottom\right)}{1+10^{\left({\left(logEC50-x\right)}^{*}slope\right)}}$$

Two-way ANOVA, followed by a *post hoc* Bonferroni test, wasused to test for differences in concentration-response curves in isolated vessel segments. The significance level was ^∗^*P* < 0.05 for all tests.

## Results

### Characteristics of the Animals

The experimental weight in male and female db/db mice was significantly higher than in db/db+ mice (Table 2), and blood glucose was markedly higher in male and female db/db mice compared to db/db+ control mice (Table 2). The blood glucose levels were not different in db/db+ male versus female mice but was higher in male versus female db/db mice. The tibia length of the normoglycaemic db/db+ male mice (1.78 ± 0.01 cm, *n* = 15) was significantly longer than in diabetic male db/db mice (1.66 ± 0.02 cm, *P* < 0.001, *n* = 11). Heart weight indexed to tibia length was unaltered and was, respectively, 94 ± 4 mg/cm (*n* = 15) and 97 ± 6 mg/cm (*n* = 11) in male db/db+ and db/db mice.

**TABLE 2:** Body weight and blood glucose levels.

	Male			Female		
	n	Body weight (g)	Blood glucose (mmol/L)	n	Body weight (g)	Blood glucose (mmol/L)
db/db+	33	29[18–36]	7.9[5.6–12.4]	33	21[10–27]#	7.4[4.3–13.8]
db/db	33	51[42–60]*	28.5[17.0–33.3]*	35	49[39–53]*	18[9.8–31.8]*

The results are represented as medians [IQR], where n indicates the number of animals examined. * P < 0.05 versus db/db+. ^#^P < 0.05 versus the same parameter in male mice in the same condition.

### Passive Properties and Collagen Content in Aorta Segments

**FIGURE 1:**
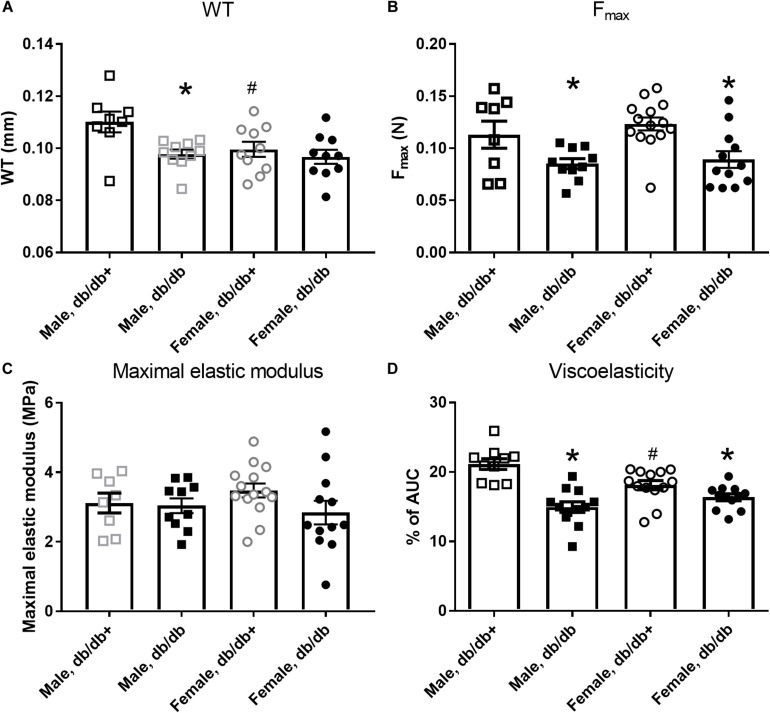
Mechanical properties of aorta segments from male and female diabetic db/db and normoglycaemic db/db+ mice. Aorta segments from diabetic db/db mice show **(A)** reduced wall thickness (WT) in segments from male db/db (*n* = 10) versus db/db+ (*n* = 8) mice, but not in female db/db (*n* = 12) and db/db+ (*n* = 14) mice and **(B)** reduced maximal load (Fmax), **(C)** unaltered maximal elastic modulus, and **(D)** reduced viscoelasticity. The results are means ± SEM. *P < 0.05, db/db versus db/db+ controls. ^#^*P* < 0.05, female versus male mice in same condition.

The passive internal diameters of the frozen and thawed aorta segments from db/db mice were, respectively, 767 ± 27 and 752 ± 26 μm in aorta segments from male db/db+ and db/db mice. Wall thickness was significantly reduced in aorta from male db/db mice (Figure 1A). Consistent with the lower wall thickness, the maximal load (Figure 1B) and stiffness (not shown) were significantly reduced. The collagen content per mm luminal circumference was reduced (Table 3), but the maximal load and stiffness normalized to collagen per mm luminal circumference (N × mm/mg, not shown) were not found to be different. This indicates that the mechanical quality of the collagen is similar in male db/db and db/db+ mice. Reduction in wall thickness only partially explains the reduction in maximal mechanical strength since maximal load and stiffness normalized to vessel wall area i.e., maximal stress (not shown) and maximal elastic modulus (Figure 1C), respectively, were not different. Cyclic testing to a low load value gave higher energy recovery for db/db mice (energy loss is less in db/db mice, Figure 1D). The distensibility of the aortic specimens at a load corresponding to 100 and 120 mm Hg (i.e., physiological pressures) was unaltered in these mice (Table 4). Thus, the aorta in male db/db mice appears to be able to accommodate more blood during systole and to recoil with less energy loss, i.e., has a better efficiency as an elastic reservoir (Windkessel). Since both the collagen and elastin percentages as well as the collagen: elastin ratio were unaltered in male db/db mice (Table 3), a reduction in the absolute collagen content (μg) also implies a reduction in absolute elastin content (μg) in db/db mice.

**TABLE 3:** Collagen per mm luminal circumference and percentage collagen and elastin of dry defatted weight.

	Male db/db+	Male db/db
Collagen (mg × 10^–3^/mm)	6.00 ± 0.49 (8)	4.42 ± 0.08* (10)
Collagen (%)	14.6 ± 0.5 (9)	13.8 ± 1.1 (10)
Elastin (%)	32.1 ± 0.9 (9)	30.8 ± 2.1 (10)
Collagen/elastin ratio	0.450 ± 0.020	0.454 ± 0.012

Means ± SEM (No. of mice). Percentages of collagen and elastin were determined for 4 pools of aortas from 2 to 3 mice. * P < 0.05 db/db versus db/db+. Collagen content for aorta from female mice is listed in the result section, while elastin content was not determined in female mice.

**TABLE 4:** Distensibility (strain) of the aortic wall at two loading pressures.

	Male db/db+ (*n* = 8)	Male db/db (*n* = 10)	Female db/db+ (*n* = 14)	Female db/db (*n* = 12)
100 mmHg	0.747 ± 0.013	0.767 ± 0.015	0.759 ± 0.021	0.862 ± 0.036*
120 mmHg	0.837 ± 0.014	0.855 ± 0.013	0.856 ± 0.019	0.955 ± 0.036*

The values are means ± SEM. * P < 0.05 db/db versus db/db+.

Passive mechanical studies were also performed in aorta segments from female db/db and db/db+ mice. The passive internal diameters of the frozen and thawed aorta segments were, respectively, 650 ± 15 and 688 ± 20 μm, in aorta segments from female db/db+ and db/db mice. In contrast to male db/db mice, the wall thickness of the aorta segments from female db/db mice was unaltered (Figure 1A). In the aorta, the maximal load was significantly reduced in female db/db mice (Figure 1B) as was the maximal stiffness (not shown). This can be explained by the reduced collagen content per mm luminal circumference from 5.67 ± 0.23 (*n* = 14) in female db/db+ to 4.48 ± 0.14 mg × 10^–3^/mm (*P* < 0.05, *n* = 12) in db/db mice. Similar to the aorta from male db/db mice maximal load and stiffness normalized to collagen per mm luminal circumference (N × mm/mg, not shown) was not found to be different. Also in contrast to male mice, the aortic segments with inactivated smooth muscle cells from female db/db mice showed increased (*p* < 0.05) distensibility (strain) at 100 and 120 mm Hg (Table 4).

**FIGURE 2:**
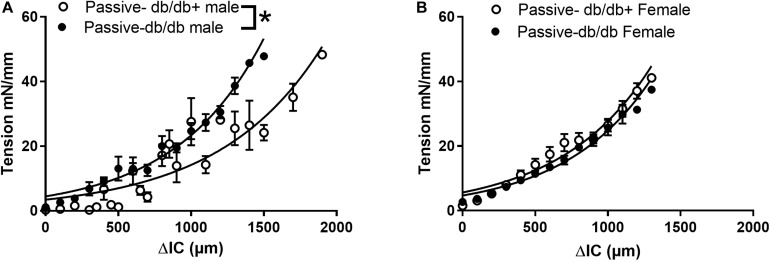
Passive and active properties of aorta segments from male and female diabetic db/db and normoglycaemic db/db+ mice. Aorta segments were mounted in myographs for isometric tension measurements allowing us to also evaluate the active vascular smooth muscle component. **(A)** Passive stretch of aorta segments measured as increase in internal circumference (ΔIC) against increase in force (ΔF) from male db/db (*n* = 6) and db/db+ mice (*n* = 6). **(B)** The passive stretch of aorta segments measured as an increase in internal circumference (ΔIC) against an increase in force (ΔF) from female diabetic db/db (*n* = 6) and control db/db+ mice (*n* = 6). The results are means ± SEM, *P < 0.05, db/db versus db/db+ controls.

In addition to the extracellular matrix, the vascular smooth muscle layer also contributes to the properties of vascular segments. Tension-stretch test was therefore performed on freshly dissected specimen in physiological saline. Successively stretching revealed increased passive tension development in wire-mounted aorta segments from diabetic db/db male mice (Figure 2A). At the optimal active tension, the aorta segments from male db/db mice had a mean internal diameter of 823 ± 53 μm (*n* = 8) which was significantly decreased compared to the diameter of segments [1144 ± 43 μm (*n* = 8)] isolated from db/db+ mice. These findings show that in the presence of active vascular smooth muscle cells, the response to passive stretch was markedly increased in aorta from male db/db mice.

In contrast, wire-mounted aorta segments from female db/db mice that were successively stretched revealed no change in passive tension development (Figure 2B). The aorta segments from diabetic female db/db mice had lumen diameters of 762 ± 91 μm (*n* = 8) at optimal tension, which was significantly decreased compared to segments isolated from db/db+ mice with lumen diameters of 1088 ± 45 μm (*P* < 0.05, *n* = 8).

### Passive Properties in Coronary Arteries From Diabetic Mice

**FIGURE 3:**
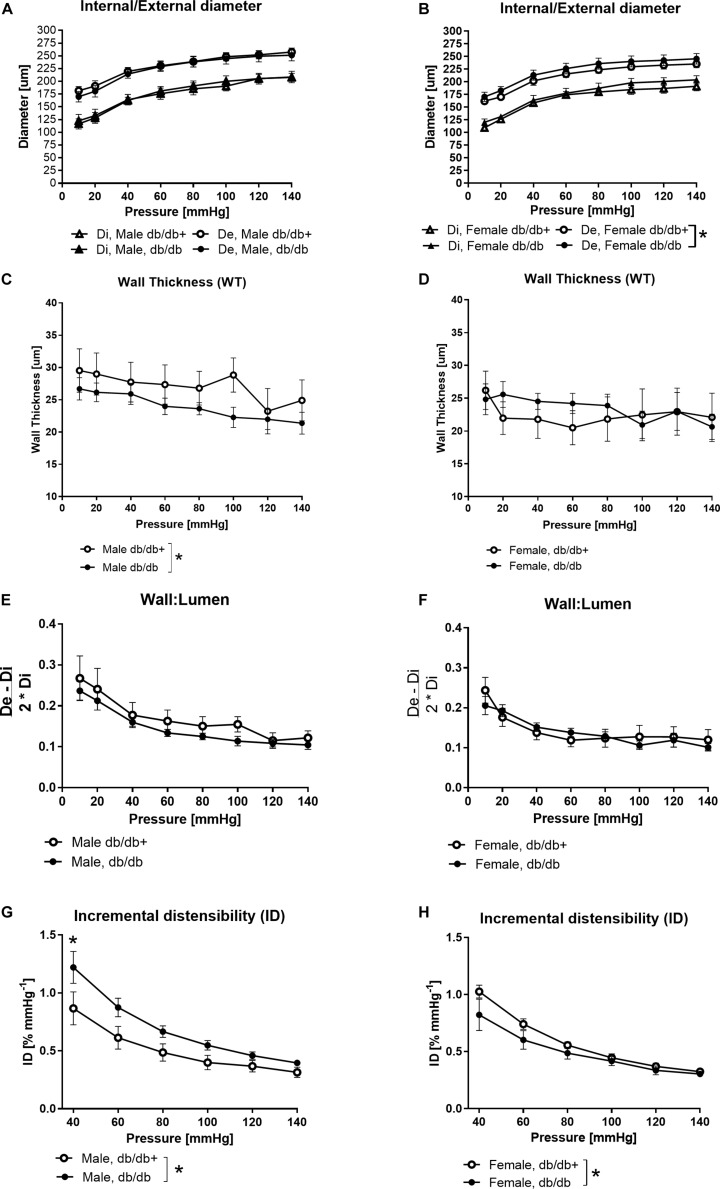
Structural and mechanical changes in left anterior descending (LAD) coronary artery from male diabetic mice. **(A,B)** Increasing pressure (10–140 mmHg) changes in external (De) and internal (Di) diameters, **(C,D)** wall thickness, **(E,F)** wall:lumen ratio, and **(G,H)** incremental distensibility. The vessel wall thickness is significantly lower in male mice, while distensibility is significantly higher in arteries from male db/db mice and significantly lower in arteries from female db/db mice compared to the db/db+ controls. The results are means ± SEM. *P < 0.05, db/db (*n* = 6) versus db/db+ controls (*n* = 6).

External diameters were slightly increased in coronary arteries form female db/db compared to db/db+ mice, but otherwise diameters of the coronary arteries from male and female db/db mice were unaltered compared to segments from db/db+ mice (Figures 3A,B). The wall thickness was significantly decreased in segments from male db/db mice (Figure 3C), while it was unaltered in coronary arteries from female db/db mice (Figure 3D). The wall to lumen ratio was unaltered (Figures 3E,F). In agreement with the decreased wall thickness, the incremental distensibility was markedly increased at low pressures in coronary arteries from male db/db mice (Figure 3G). In contrast, incremental distensibility was decreased in coronary arteries from female db/db arteries compared to age-matched db/db+ mice (Figure 3H).

**FIGURE 4:**
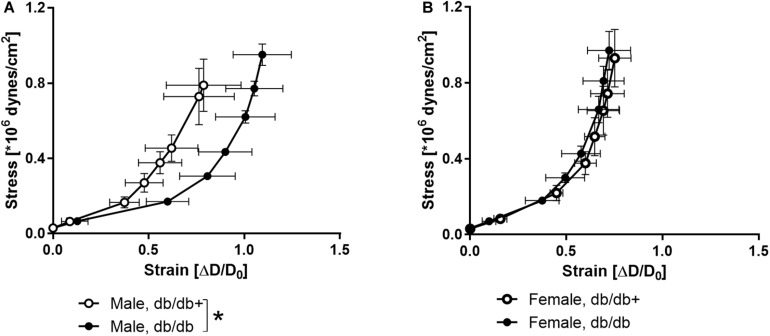
Stress-strain curves in coronary arteries. Strain measured as change in diameter (ΔD) over initial vessel diameter (D_0_) plotted against stress in coronary arteries from **(A)** db/db+ and db/db male mice and **(B)** db/db+ and db/db female mice. *P < 0.05, db/db (*n* = 6) versus db/db+ controls (*n* = 6).

The stress-strain curve was rightward shifted indicating decreased stiffness in coronary arteries from male db/db mice (Figure 4A), while the stress-strain curve of coronary arteries from female db/db mice was unaltered (Figure 4B).

### Functional Studies in the Aorta and Coronary Arteries

At the optimal passive tension, the active tension responses to 60 mM KPSS were not significantly altered in aorta segments from female db/db+ and db/db mice were, respectively, 5.5 ± 1.1 Nm^–1^ (*n* = 12) and 4.4 ± 0.4 Nm^–1^ (*n* = 10), and in aorta segments from male db/db+ and db/db mice, respectively, 7.5 ± 0.5 Nm^–1^ (*n* = 11) and 6.6 ± 0.8 Nm^–1^ (*n* = 11).

**FIGURE 5:**
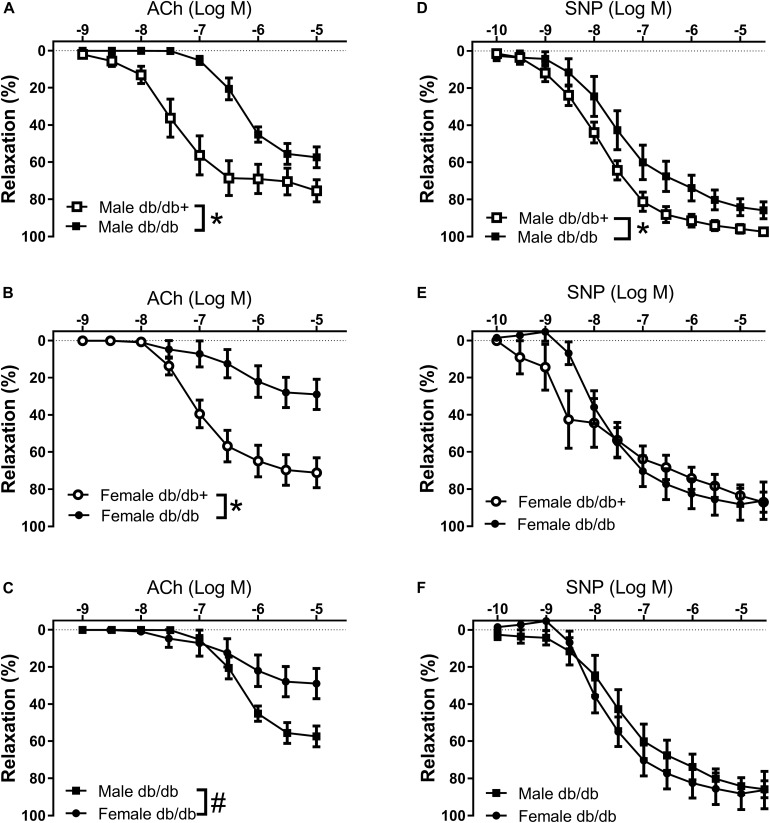
Impaired endothelium-dependent relaxation in aorta from diabetic db/db mice. Average aorta relaxations in segments contracted with phenylephrine. **(A)** Impaired acetylcholine (ACh) relaxation in diabetic db/db male mice (*n* = 6) compared to normoglycaemic db/db+ male mice (*n* = 6). **(B)** Impaired acetylcholine relaxation in diabetic db/db female mice (*n* = 6) compared to normoglycaemic db/db+ (*n* = 7) female mice. **(C)** The impairment of acetylcholine relaxation was more pronounced in aorta segments from female compared to male diabetic db/db mice. **(D)** Impaired SNP relaxation in aorta from diabetic db/db male mice (*n* = 6) compared to normoglycaemic db/db+ (*n* = 7) male mice. **(E)** Unaltered relaxations induced by the NO donor sodium nitroprusside (SNP) in diabetic db/db female mice (*n* = 6) compared to normoglycaemic db/db+ female mice (*n* = 6). **(F)** SNP relaxations were similar in aorta segments from male and female db/db mice. The results are means ± SEM. *P < 0.05 db/db versus db/db+ control mice. ^#^*P* < 0.05, female versus male mice in the same condition.

The arteries were contracted with 10^–7^ M phenylephrine, which induced comparable contractions in the aorta segments (Table 5). Phenylephrine-contracted segments relaxed to acetylcholine, but the acetylcholine relaxation curves were significantly reduced in aorta segments from male and female db/db mice (Figures 5A,B and Table 5). Comparing acetylcholine relaxations in aorta segments from male versus female control db/db+ mice showed the concentration-relaxation curves were not different, while acetylcholine relaxations were significantly reduced in aorta segments from female versus male db/db mice (Figure 5C and Table 5). These findings suggest the impairment of relaxations to the endothelium-dependent vasodilator, acetylcholine, is more pronounced in aorta segments from female db/db mice.

**TABLE 5:** Relaxations induced by acetylcholine (Ach) and sodium nitroprusside (SNP) in aorta segments from male (M) and female (F) normoglycaemic (db/db+) and diabetic (db/db) mice.

	PhE	ACh		PhE	SNP	
	Nm^–1^	-log(EC_50_)	Max relaxation (%)	Nm^–1^	-log(EC_50_)	Max relaxation (%)
M db/db+	2.2 ± 0.5	7.47 ± 0.15	72.3 ± 4.3	2.5 ± 0.5	7.93 ± 0.08	96.0 ± 2.2
M db/db	2.3 ± 0.6	6.34 ± 0.07*	57.9 ± 3.1*	3.7 ± 0.9	7.49 ± 0.17	84.3 ± 4.5*
F db/db+	1.7 ± 0.2	7.07 ± 0.12	69.8 ± 4.2	1.7 ± 0.4	7.83 ± 0.12	85.2 ± 3.7^#^
F db/db	1.8 ± 0.4	6.38 ± 0.45	31.5 ± 9.6*^#^	2.3 ± 0.2	8.21 ± 0.20	76.9 ± 3.7

*Aorta segments were contracted with phenylephrine (PhE, tension in Nm^–1^). EC_50_ is the concentration causing half-maximal relaxation. The data are means ± SEM of n = 6–7 animals. ^∗^P < 0.05, Students t-test versus the same parameter in aorta segments from db/db+ mice. ^#^P < 0.05, Students t-test versus same parameter in male mice of same strain.*

The NO donor SNP induced concentration-dependent relaxations, which were significantly reduced in aorta segments from male db/db mice (Figure 5D). These findings suggest endothelial dysfunction and reduced smooth muscle response to a NO donor in aorta from male db/db mice. Relaxations induced by the NO donor SNP were unchanged in aorta segments from female db/db mice (Figure 5E). Comparing SNP relaxations in aorta segments from male versus female db/db + showed maximum relaxations were less in segments from female mice (Table 5) while comparing male and female db/db mice showed that the concentrations-response curves were not different.

In addition to a sex-specific difference in vascular function, the age and most likely glucose exposure time appears to play a role. Another series of mice, showed that in 11-week-old male db/db mice there was only a small rightward shift in the concentration-response curves for acetylcholine and no changes in the SNP relaxations comparing aorta from db/db versus control db/db+ mice (Supplementary Figure S2); and while there were no difference in responses to acetylcholine and SNP in the aorta from 11-week versus 16-week old db/db+ mice, both acetylcholine and SNP relaxations were diminished in the aorta from 16-week-old db/db mice (Supplementary Figure S2).

In wire-mounted coronary arteries, the internal diameters were, respectively, 211 ± 12 μm (*n* = 6) and 210 ± 12 μm (*n* = 7) from male diabetic db/db and db/db+ mice. In the coronary arteries of db/db mice contracted with U46619, acetylcholine relaxation was impaired in arteries from male diabetic mice compared to db/db+ control mice (Supplementary Figure S3A). The relaxations induced by the NO donor, SNP, were significantly reduced in coronary arteries from db/db mice (Supplementary Figure S3B).

### Blood Pressure and Transthoracic Echocardiography

Mean arterial blood pressure measured in anesthetized male mice was not different in male diabetic db/db compared to db/db + control animals (Table 6), but the heart rate was lower. The duration of the PR-interval, QT-interval and the corrected QT-interval on the ECG were significantly prolonged in diabetic animals (Table 6 and Supplementary Figure S4). The duration of P-wave was not significantly different from the controls, however, therefore the prolonged PR-interval was due to prolonged duration of the PR-segment (43.2 ms vs. 37.3 ms, *p* = 0.035).

**TABLE 6:** Mean arterial pressure (MAP) and echocardiographic results in a subset of male control db/db + and diabetic db/db mice.

	db/db+ (*N* = 7)	db/db (*N* = 7)	*p*
MAP (mmHg)	73 ± 5	68 ± 2	0.38
Heart rate (min^–1^)	400 ± 17	340 ± 11	0.01*
Ejection time (ms)	54.8 ± 2.8	65.2 ± 2.8	0.02*
Duration of diastole (ms)	96.6 ± 4.6	112.4 ± 5.8	0.05
p-wave duration (ms)	13.5 ± 0.7	14.7 ± 0.8	0.26
PR-interval (ms)	50.7 ± 1.5	58.0 ± 1.5	0.01*
QT interval (ms)	50.5 ± 1.5	64.0 ± 3.0	0.001*
QTc (ms)	41.2 ± 1.3	48.0 ± 1.4	0.004*
LVPW_d_ (mm)	1.01 ± 0.07	1.11 ± 0.08	0.39
LVPW_d_/TL (mm/cm)	0.57 ± 0.04	0.67 ± 0.05	0.14
LVPW thickening (%)	61.4 ± 8.1	37.8 ± 2.2	0.03*
LVID_d_ (mm)	3.59 ± 0.09	3.92 ± 0.09	0.03*
LVID_d_/TL (mm/cm)	2.02 ± 0.05	2.36 ± 0.05	<0.001
LVarea_d_ (mm^2^)	8.89 ± 0.37	11.2 ± 0.45	0.002*
LV area_d_/TL (mm^2^/cm)	5.00 ± 0.21	6.72 ± 0.27	< 0.001*
Fractional foreshortening (%)	42.3 ± 3.0	37.5 ± 1.9	0.20
Ejection fraction (%)	79.8 ± 3.1	75.1 ± 2.1	0.23
Fractional area change (%)	65.7 ± 2.5	58.8 ± 2.9	0.10
Aorta ID_d_ (mm)	1.28 ± 0.05	1.27 ± 0.03	0.90
Aorta ID_d_/TL (mm/cm)	0.72 ± 0.03	0.72 ± 0.02	0.90
Circumferential strain (%)	23.1 ± 2.4	17.8 ± 4.3	0.30
Aortic V_max_ (mm/s)	1008 ± 110	655 ± 76	0.02*
Aortic V_mean_ (mm/s)	581 ± 63	379 ± 43	0.02*
Stroke volume (mm^3^)	29.2 ± 2.5	29.7 ± 3.4	0.91
Stroke volume/TL (mm^3^/cm)	16.4 ± 1.43	16.7 ± 1.92	0.91
Cardiac output (ml/min)	11.5 ± 0.7	10.0 ± 1.1	0.28
Cardiac output/TL (ml/min/cm)	6.46 ± 0.39	5.63 ± 0.62	0.28

BM: body mass, d: end-diastole, ID: inner diameter, LV: left ventricle, PW: posterior wall, TL: tibia length, V: velocity. The values are means ± SEM. * P < 0.05, versus db/db+ (Student’s t-test).

**FIGURE 6:**
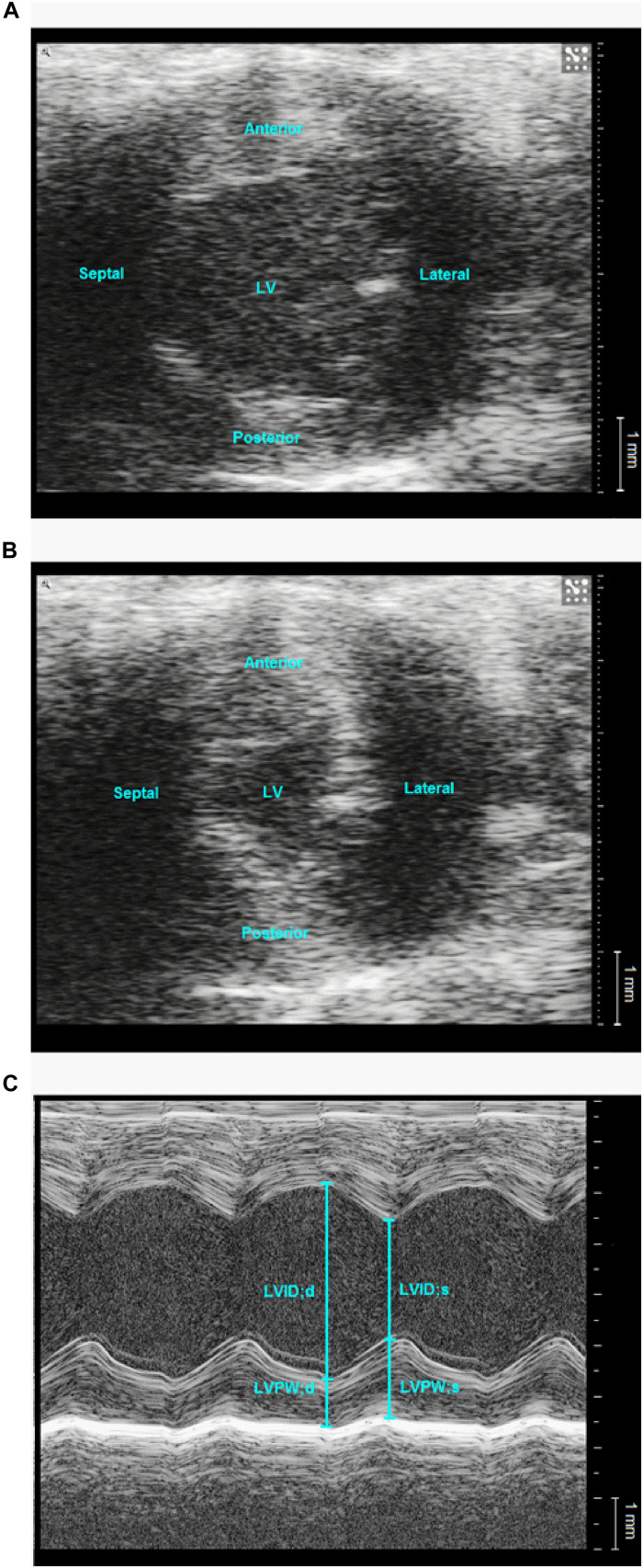
Representative echocardiographic images of left ventricle in a normoglycaemic db/db+ mouse. Representative B-mode **(A,B)** and M-mode **(C)** images of the left ventricle (LV) at mid-papillary level from the parasternal short axis view. LV area was measured at end-diastole **(A)** and end-systole **(B)**. d: diastole, LVID: left ventricular inner diameter, LVPW: left ventricular posterior wall, s: systole.

Left ventricle dimensions were measured (Figure 6), and LV cavity size, both the absolute size and per centimeter tibia length, of the db/db mice was significantly greater than the heterozygote mice (Table 6). LV posterior wall thickening, fractional foreshortening, ejection fraction and fractional area change were lower in the db/db mice. However, only the difference in the posterior wall thickening was statistically significant. The stroke volume was similar in the two groups of mice and the cardiac output was mildly reduced (no statistical significance) as a result of lower heart rate. Echocardiography of aorta showed decreased mean flow and maximal flow velocity in diabetic db/db mice (Table 6 and Supplementary Figure S5). The circumferential strain of the ascending aorta was lower in the db/db mice, but the difference did not reach statistical significance.

## Discussion

In this comprehensive study we have examined in detail the passive and active changes in the mechanical and functional properties of the large arteries as well as the alterations in cardiac function and hemodynamics in diabetic mice. In addition, we investigated the impact of sex and age on the mechanical and functional properties of the arteries in diabetic mice.

We observed a structural adaptation of the heart and vasculature to increased body weight, increased blood glucose and decreased locomotion in the diabetic animals. Consistent with a reduced vessel wall thickness and collagen content in the aorta of male db/db mice, we observed a reduced maximal load and decreased viscoelasticity indicative of a more efficient Windkessel function. Similar findings were observed in the coronary artery. However, testing the arterial segments with preserved vascular smooth muscle function revealed increased vasomotor tone and endothelial dysfunction in diabetic male mice. This was evidenced by increased tension development to stretching and impaired relaxation to acetylcholine and sodium nitroprusside and the impairment was more pronounced in older diabetic male mice. Sex-specific vascular changes were also observed. While the decrease in maximum load and viscoelasticity of the aorta in female diabetic mice was comparable to that of the male mice, the impairment to acetylcholine relaxation was more pronounced in female mice. Moreover, in contrast to diabetic male mice, the incremental distensibility of the coronary artery decreased in diabetic female mice.

### Vascular Remodeling

Macroangiopathy associated with the development of accelerated atherosclerosis plays an important role in the development of vascular complications in type 2 diabetic patients (Di Marzio et al., 2006). The findings regarding aortic remodeling in diabetic mouse models are more controversial. In aorta from diabetic ob/ob mice, the media thickness is increased and this is ascribed to an increased amount of proteoglycans in the vessel wall (Okon et al., 2003), while in db/db mice, wall thickness of the aorta was reported to be similar to that of the control mice (Guo et al., 2005). *In vitro* studies reported larger diameters of stretched aorta segments from db/db mice (Piercy and Taylor, 1998), but recent *in vivo* studies suggested similar aortic diameters measured with echocardiography (Faita et al., 2018). In the present study, the diameters of the aorta in db/db versus control mice were similar using echocardiography, and in segments without active contribution from smooth muscle cells. In segments with active smooth muscle cells, the diameter of aorta segments from db/db male mice was smaller suggesting a larger smooth muscle response to stretch. Moreover, wall thickness was decreased in aorta from male db/db mice. These findings suggest that mean arterial diameters are similar in aorta from db/db and control mice, but that an adaptation to a lower flow velocity with thinning of the vessel wall and change in the passive properties have taken place in the aorta from db/db mice (Figure 1). Apart from the hyperglycemia, the db/db mice due to the leptin receptor defect have pronounced obesity which may also contribute to these alterations.

In patients with type 2 diabetes, a hypertrophic remodeling with larger media-to-lumen ratio take place in small subcutaneous arteries (Rizzoni et al., 2001; Schofield et al., 2002). Mesenteric small arteries, septal coronary arteries, and coronary microvessels from db/db mice also have increased media-to-lumen ratio and smaller lumen diameters consistent with changes observed when hypertrophic inward remodeling takes place (Katz et al., 2011; Anghelescu et al., 2015; Husarek et al., 2015; Nguyen Dinh Cat et al., 2018). Hypertrophic outward remodeling has also been suggested to occur in even smaller (internal lumen diameters∼50 μm) mesenteric arteries from db/db mice (Souza-Smith et al., 2011). In the present study, similar experimental setups did not reveal any differences between the inner and outer diameters of the left anterior descending coronary artery from male db/db mice compared to control db/db+ mice. The inner diameters of the arterial segments in the present study are similar to previous studies on the mouse septal coronary artery (Katz et al., 2011; Husarek et al., 2015). However, taking the dilation of the left ventricle observed with echocardiography in db/db mice into consideration, it cannot be excluded that the longitudinal stretch of the LAD may contribute to increased resistance and reduced coronary blood flow in the diabetic mouse heart, as previously observed in the male db/db mice (Husarek et al., 2015).

### Arterial Stiffness

Arterial stiffening is considered an independent risk factor for both macro-and micro-vascular complications in type 2 diabetes (Schram et al., 2004; Prenner and Chirinos, 2015). Aortic stiffness is markedly increased in the diabetic Zucker rat and is attributed to increased fibronectin and collagen IV (Sista, 2005). In the present study, the maximum stiffness of the aorta was decreased in the male db/db mice consistent with the decreased wall thickness, while the maximal elastic modulus remained the same. The aortic stiffness evaluated *in vivo* also appeared unchanged consistent with a recent study (Faita et al., 2018). The stress-strain relationship was rightward shifted in coronary arteries from male db/db mice. These findings agree with previous findings of decreased stiffness in mouse septal coronary arteries (Katz et al., 2011; Husarek et al., 2015), but contrast markedly with previous studies reporting increased stiffness in mesenteric arteries (Nguyen Dinh Cat et al., 2018). These findings suggest that the changes are related to the vascular bed affected in the db/db mice, and in the mesenteric bed, vascular smooth muscle or perivascular formation of aldosterone may contribute to the development of changes in the arterial function and structure (Briones et al., 2012; Matsuzawa et al., 2013). Although there is an increase in subcutaneous fat accumulation, in the db/db mice, fat accumulation primarily takes place around the viscera and there is less accumulation in the thorax and around the coronary arteries that are embedded in the myocardium, hence allowing less access from fat-derived autacoids than in the mesenteric vascular bed.

### Arterial Viscoelasticity

In large arteries from diabetic patients, elasticity was reported to be decreased (Airaksinen et al., 1993). Septal coronary arteries from db/db mice exhibit decreased elastic modulus, while incremental elastic modulus was increased in the femoral arteries of the same mice (Katz et al., 2011). In the present study, the viscoelastic behavior was decreased in aorta from both female and male db/db mice as well as in coronary arterioles from male mice (Figure 2). This can be explained by compositional changes in the content of collagen, elastin, glycosaminoglycans (GAG), and/or smooth muscle cells. We found a decreased collagen content and unaltered fractional elastin content in the aorta from male db/db mice. In the aorta, engagement of elastic fibers plateau around a strain of 0.2 (Chow et al., 2014), while at physiological pressures, aorta operates at a higher strain (>0.5), and therefore requires substantial recruitment of collagen fibers. Therefore, the reduced collagen content will lead to decreased viscoelasticity, i.e., less energy dissipation to pressure waves. It may also contribute to the decrease in maximal load at a breaking point in the aorta segments from both female and male diabetic db/db mice.

### Vascular Contractility

Several studies have found increased contractility both to high extracellular K^+^ and to alpha-adrenoceptor agonists in the aorta and small arteries from diabetic rats and mice (Piercy and Taylor, 1998; Pannirselvam et al., 2002, 2005; Xie et al., 2006; Sallam et al., 2011). In several of these studies of aorta from db/db mice, the passive tension in segments from db/db and db/db+ mice were set to a fixed passive tension load, e.g., 5.5 mN, corresponding to 30 mmHg, or 1.5 g (Piercy and Taylor, 1998; Miike et al., 2008; Sallam et al., 2011). In the present study, the arterial segments were stretched for optimal tension development, and at this level the active responses were similar. However, the myogenic response to stretch was increased in aorta from male db/db mice, which is in agreement with previous studies demonstrating increased contractility in aorta from male db/db mice. In contrast, we found that the active responses to stretch were similar in aorta segments from db/db and control female mice. These findings suggest that sex play a role for the augmentation of smooth muscle contractility in the aorta from male db/db mice.

Reduced relaxations to the NO donor, SNP also suggest alterations of smooth muscle function diabetes. Thus, endothelium-independent concentration-dependent relaxations to the NO donor SNP were unaltered in the aorta of 6–8-week-old mice, rightward shifted in the aorta of 11 weeks old male db/db mice (Miike et al., 2008), and markedly reduced in the aorta from 14 to 20 weeks old male db/db mice (Sallam et al., 2011). We also observed that SNP-induced endothelium-independent vasodilatations were markedly impaired in the aorta and coronary arteries from 16-week old male diabetic db/db mice, while there was no change in SNP relaxations of aorta from 11-week old male db/db mice. These observations suggest that long-term exposure to high glucose levels lead to impairment of SNP relaxations in aorta from male db/db mice. In contrast to the male db/db mice, SNP relaxations in aorta from female db/db mice were unaltered suggesting that the smooth muscle dysfunction is most pronounced in aorta from male db/db mice. The diabetic female mice had smaller body weight and less increase in plasma glucose compared to male mice, and this may contribute to the observations that structural and aorta contractility alterations appear to be less pronounced in aorta from diabetic female mice compared to diabetic male mice.

### Endothelial Dysfunction

Numerous studies have shown endothelial dysfunction in patients with type 2 diabetes (Shi and Vanhoutte, 2017) and in mouse coronary arterioles (Gao et al., 2007; Zhang et al., 2008; Choi et al., 2012; Kassan et al., 2014) and aorta segments from db/db mice (Piercy and Taylor, 1998; Miike et al., 2008; Sallam et al., 2011). Acetylcholine relaxation is unaltered in aorta of 6-week old male db/db mice (Miike et al., 2008), but impaired endothelium-dependent acetylcholine relaxation is present in 6–8-week old male db/db mice which have higher glucose levels and subsequently progresses with further reduction of acetylcholine relaxation in aorta of db/db mice at 11 weeks (Miike et al., 2008) and 14–20 weeks (Sallam et al., 2011). In aorta from male db/db mice, we also observed an age-dependent progression in the impairment of acetylcholine relaxation. In addition, the impairment of acetylcholine relaxation was significantly reduced in the aorta of female db/db mice, not only compared to aorta from control animals, but also compared to the aorta from male db/db mice. The blood glucose level *per se* is unlikely to explain the difference in impaired endothelial function compared to male diabetic db/db mice, as the levels were lower in female versus male db/db mice (Table 2). Sex hormones affect the cardiovascular system, but comparing arteries from male and female animals often show that estrogens are protective and improve endothelial function (Wellman et al., 1996), but this is opposite to the present findings of decreased acetylcholine relaxation. Weight and inflammation were suggested as the major determinants of vascular dysfunction in the aorta of male db/db mice (Sallam et al., 2011). Expressed relative to the control animals, the weight gain in female db/db mice is markedly larger than in male db/db mice in the present study, which would agree with weight being important for endothelial dysfunction. Although further investigation will be required to clarify the underlying mechanism, our results may contribute to understand the observation of higher incidence of macrovascular complications in diabetic women compared to diabetic men (Maric-Bilkan, 2017).

### Heart Rhythm and Function in Diabetic db/db Mice

The heart rate of the animals, both db/db+ and db/db mice, is low. This is likely attributed to full anesthesia. In contrast to diabetic human subjects (Ewing et al., 2015), there was a fall in heart rate in the diabetic mice, which is consistent with previous observations (Park et al., 2008; Senador et al., 2009). This could be due to reduced locomotor activity and metabolism (Park et al., 2008) and/or altered autonomic balance in the diabetic mouse hearts (Senador et al., 2009). Mice can increase their heart rate by only 30–40%, while humans can increase the heart rate by ∼300% (Kaese and Verheule, 2012). Therefore, increasing the heart rate will only lead to a small increase in stroke volume and cardiac output in mice. In contrast, decreased heart rate, i.e., longer cardiac duration with a longer diastolic filling time, will lead to an increase in ventricular preload resulting in increased ventricular contractility and stroke volume as dictated by Starling’s law. This may partially explain the observed decrease in heart rate in diabetic mice. Diabetes is associated with an increased risk of impaired electrical conduction in the heart, due to autonomic neuropathy in diabetic patients. Defects such as atrioventricular block, bundle branch block, and prolonged QT/QTc, will correlate to incremented cardiovascular risk factors, leading to increased mortality rates (Movahed, 2007). In diabetic mice, the prolonged PR-interval indicates impaired atrioventricular conduction. We also observed a wide M-shaped QRS-complex, and the QRS complex is composed of not only depolarization but also early repolarization in mice (Boukens et al., 2014). A prolonged QRS could be due to prolonged depolarization or early repolarization of the ventricles resulting in a longer ejection time in the diabetic mice, which seems like a further functional compensatory effect of the diabetic autonomic neuropathy.

Left ventricle systolic function in db/db mice has previously been reported to be impaired (Plante et al., 2014; Baumgardt et al., 2016; Moon et al., 2016; Xu et al., 2016). Although different types of control animals (C57BL6 or db/db+) have been included, and db/db mice were investigated at different ages, reduced stroke volume, foreshortening fraction, ejection fraction, and cardiac output values have been reported and seem to develop with increasing age of the animals (Plante et al., 2014; Baumgardt et al., 2016; Moon et al., 2016; Xu et al., 2016). In the present study, the LV systolic function of the db/db mice was reduced as evidenced by the decreased posterior wall thickening, fractional foreshortening, ejection fraction and fractional area change, although the reduction in the three latter parameters did not reach statistical significance. Due to the prolonged diastolic filling time and ejection time, the stroke volume was maintained and the cardiac output was only mildly reduced (statistically insignificant) as a consequence of lower heart rate.

Diabetic cardiomyopathy can occur in the hypertrophic and/or the dilated form or as restrictive cardiomyopathy in diabetic patients with preserved ejection fraction (Seferoviæ and Paulus, 2015). In db/db mice, a recent study observed low LV mass and systolic dysfunction suggesting restrictive cardiomyopathy rather than hypertrophic remodeling of the heart in db/db animals (Faita et al., 2018). In the present study, heart weight was unaltered expressed as raw weight and as a ratio to tibia length. Moreover, we did not observe any significant increase in the posterior wall thickness, while the LV cavity was significantly larger in the diabetic db/db mice suggestive of LV dilatation. The presence of hypertension was suggested to play a role in the cardiac volumes in db/db mice (Van Bilsen et al., 2014), but in a subset of animals, we did not find differences in mean arterial pressures measured with an invasive catheter. Although tail blood pressure measurements suggested that db/db mice are hypertensive (Bagi et al., 2005; Van Bilsen et al., 2014), our findings are in agreement with other studies reporting no difference in telemetric blood pressure values (Su et al., 2008; Katz et al., 2011; Souza-Smith et al., 2011; Husarek et al., 2015; Faita et al., 2018). The increased end-diastolic LV cavity dimensions may also be related to a longer diastolic filling time, and hence adaptation of the heart to larger body weight and reduced locomotor activity.

### Limitations

The experiments on arteries from both control db/db+ and diabetic db/db male and female mice were conducted with the same glucose concentration of 5.5 mM. This was to avoid acute hyperglycaemic effects on endothelial function (Bangshaab et al., 2019), which may confound the relation of endothelial to structural function. However, both the male and female db/db mice were exposed to high plasma glucose concentrations for several weeks. We cannot exclude the impairment of endothelial function would be more pronounced in the presence of high glucose levels in the bath solution when arteries from db/db mice were examined, and this may also influence the observed sex differences.

There are several echocardiographic studies in male db/db mice (Plante et al., 2014; Baumgardt et al., 2016; Moon et al., 2016; Katare et al., 2018), and in the present study, we also chose to focus on the male db/db mice to clarify whether the pronounced vascular changes can be explained by changes in cardiac function and aortic flow. Despite measurements of a series of parameters, our results only reflect one-time point in the development of alterations in db/db mice, and due to low animal availability, there is a lack of some of the parameters in female db/db mice in the present study.

## Conclusion and Perspectives

The vessel thickness and viscoelasticity were decreased in aorta from diabetic male db/db mice, and the aortic Windkessel function seems more efficient. Together with the observed left ventricular dilatation and reduction in aortic flow velocity, these findings suggest a structural adaptation of the heart and vasculature to the increased body weight and decreased locomotion in these animals. Sex-specific differences are apparent in vessel segments with smooth muscle contribution as the passive stretch-tension curve was leftward-shifted in the aorta from male db/db mice, while it was unaltered in female db/db aorta segments versus control db/db+ mice. The reduced relaxations to the NO donor, SNP also suggest alterations of smooth muscle function in the aorta from male diabetic db/db mice. In the aorta from female diabetic db/db mice, acetylcholine relaxation was significantly impaired compared to the aorta from both the control and male diabetic db/db mice. The findings that impairment of vascular endothelial function was most pronounced in the aorta from female diabetic mice may have implications for the observed higher incidence of macrovascular complications in diabetic women.

## Data Availability Statement

The datasets generated for this study are available on request to the corresponding author.

## Ethics Statement

The animal study was reviewed and approved by Danish Animal Experiments Inspectorate (permission 2014-15-2934-01059).

## Author Contributions

LB, EP, RC, and RH performed the functional examination of vessel segments *in vitro*. LB, RC, and CD examined the passive properties of the vessel segments. JS performed the transthoracic echocardiographic examination. SC-S performed the blood pressure measurements. All authors contributed to the design of the study and in the analysis of the data. LB, JS, and US wrote the first draft of the manuscript. All authors revised and approved the final version of the manuscript.

## Conflict of Interest

The authors declare that the research was conducted in the absence of any commercial or financial relationships that could be construed as a potential conflict of interest.

## Supplementary Material

FIGURE S1 Representative image of an aorta ring segment.

FIGURE S2 Acetylcholine and sodium nitroprusside relaxations in the aorta from 11- and 16-week old control (db/db+) and diabetic male mice.

FIGURE S3 Impaired acetylcholine and sodium nitroprusside relaxations in coronary arteries contracted by the thromboxane analog, U46619.

FIGURE S4 Representative electrocardiogram from a diabetic db/db mouse and a control db/db+ mouse.

FIGURE S5 Representative echocardiographic images of aorta in a db/db mouse.

TABLE S1 Calculation of passive properties of coronary arteries.
