# Supplementary material for: Endothelial Dysfunction and Passive Changes in the Aorta and Coronary Arteries of Diabetic db/db Mice
Source: Front Physiol. 2020 Jun 23;11:667. doi: 10.3389/fphys.2020.00667 (PMC7324802; doi:10.3389/fphys.2020.00667)
Supplement: FIGURE S1 — Representative image of an aorta ring segment. [file Data_Sheet_1.pdf]

Supplementary data file

Endothelial dysfunction and passive changes in the aorta and coronary arteries of diabetic db/db mice

Lilliana Beck[1], Junjing Su[1], Simon Comerma-Steffensen[1], Estéfano Pinilla[1], Rune Carlsson[1], Raquel Hernanz[1, 2], Majid Sheykhzade[3], Carl Christian Danielsen[1] and Ulf Simonsen[1, #]

[1]Department of Biomedicine, Pulmonary and Cardiovascular Pharmacology, Faulty of Health, Aarhus University, Denmark

[2]Departamento de Ciencias Básicas de la Salud, Universidad Rey Juan Carlos, Alcorcón, Spain

[3]Department of Drug Design and Pharmacology, Faculty of Health and Medical Sciences, University of Copenhagen, Denmark

1 Methods and materials

1.1. Animals and preparation of samples

Male and female 8-week old db/db mice (C57BLKS/J-lepr^{db}/lepr^{db}) and age- and sex-matched db/db+ littermate controls (C57BLKS/J-lepr$^{db/db+}$) were purchased from Taconic Europe (Ry, Denmark). All animal care and experimental protocols in this study were conducted under the supervision of a veterinarian and in accordance with the Danish legislation of animal use for scientific procedures as described in the 'Animal Testing Act' (Consolidation Act No. 726 of 9 September 1993 as amended by Act No. 1081 of 20 December 1995) and approved by the Danish Animal Experiments Inspectorate (permission 2014-15-2934-01059).

1.2. Transthoracic echocardiography

Echocardiography (Vevo2100, Visual Sonics, Toronto, Canada) was performed using a linear array probe (MS 550D, 22 – 55MHz) on the spontaneously breathing mouse that was placed in the left lateral decubitus position on a heating pad adjusted to 37 °C. The animal's electrocardiography (ECG) signal was captured through copper electrodes on the heating pad.

1.3. Passive mechanical studies in aorta segments

Mechanical testing was performed on aorta segments. The ring specimens were pushed at minimal strain onto a tapered glass rod until the rod filled the lumen. These were then photographed twice using a Nikon microscope equipped with a circular polarization filter. Average wall thickness was estimated from traced (ImageJ 1.50e) cross-sectional areas at four random locations. An image of a millimeter scale was used for calibration (Supplementary Figure S1).

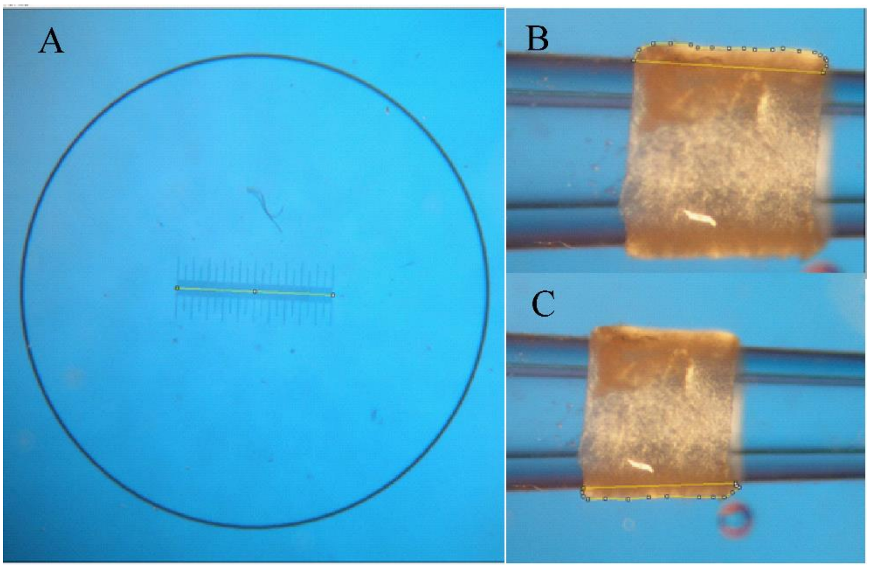

Supplementary Figure S1. Representative image of aorta ring segment. The vessel wall area (outlined in yellow) was measured by calibrating it to a millimeter scale and the vessel wall thickness was derived by assuming a height of 1 mm for all segments.

Supplementary Table S1. Calculation of passive properties of coronary arteries mounted in a small vessel pressure myograph.

**1- WT**	**Wall Thickness**	**WT** = $\frac{(D_e - D_i)}{2}$
**2- W:L**	**Wall:Lumen ratio**	**W: L** = $\frac{(D_e - D_i)}{2 \cdot D_i}$
**3- ID**	**Incremental Distensibility**	**ID** = $\frac{\Delta D_i}{D_i \cdot \Delta P} \cdot 100$
**4- σ**	**Stiffness: Circumferential wall stress**<br>**Stress-strain relationship**	$\sigma = \frac{(P \cdot D_i)}{2 \cdot WT}$
**5- ε**	**Circumferential wall strain**	**ε** $\frac{(D_i - D_{i@10mmHg})}{D_{i@10mmHg}}$
**6- σ**	**Stiffness**<br>incremental elastic modulus , $E_{inc} = \frac{\delta\sigma}{\delta\varepsilon}$	$\sigma = \sigma_{10mmHg} e^{\beta\varepsilon}$

De, external diameter; Di, internal diameter; ΔP, increase in pressure.

2 Results

2.1. Functional studies in the aorta and coronary arteries from male db/db mice

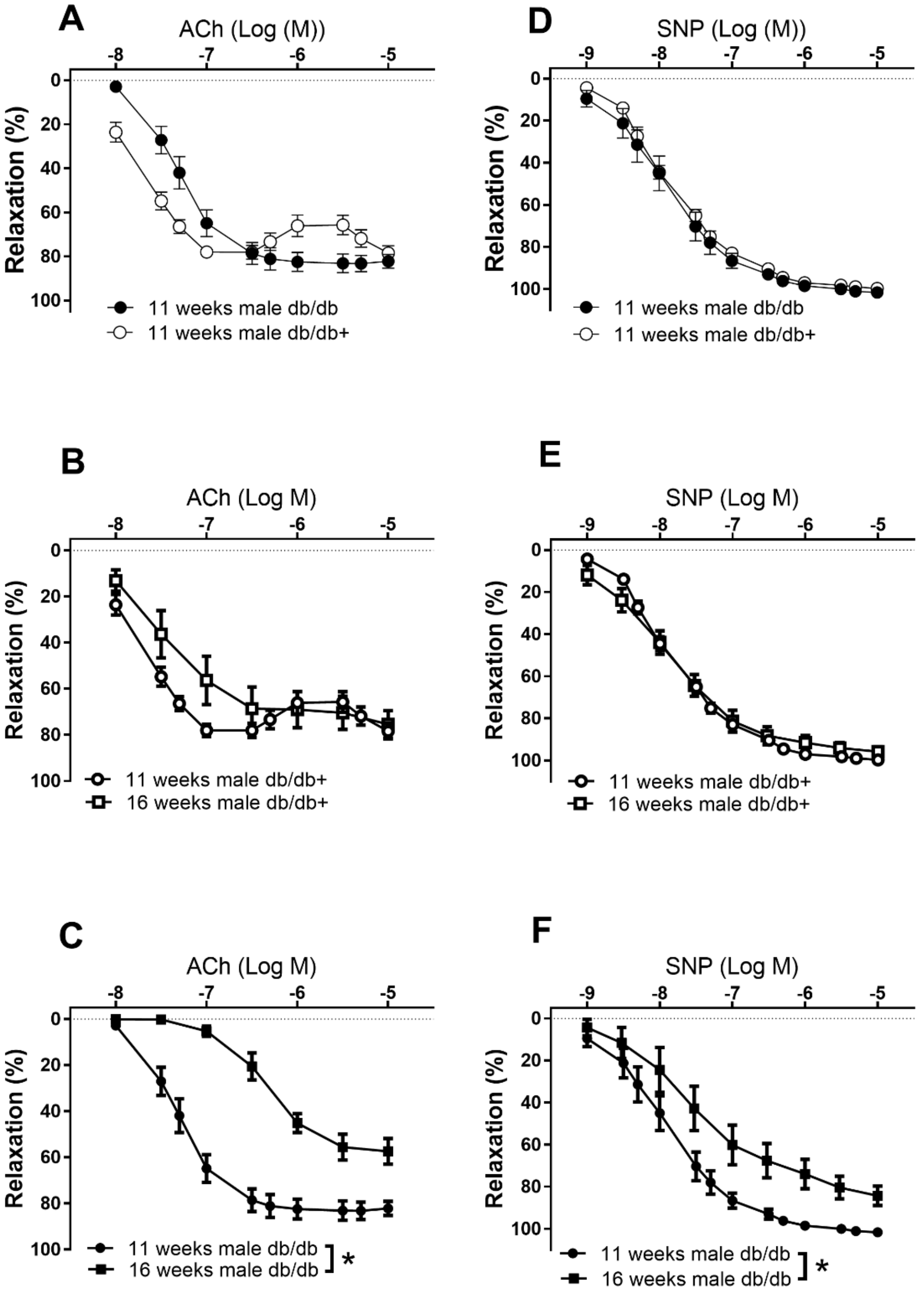

Supplementary Figure S2. Acetylcholine and sodium nitroprusside relaxations in the aorta from 11- and 16-week old control (db/db+) and diabetic male mice. Average aorta relaxations in segments contracted with phenylephrine. (A) Small rightshift in acetylcholine (ACh) relaxation in aorta from diabetic db/db male mice (n=12) compared to normoglycaemic db/db+ male mice (n=12). (B) ACh relaxation in aorta from 11-and 16-week db/db+ male mice. (C) The impairment of acetylcholine relaxation was more pronounced in aorta segments from 16-week male db/db mice

compared to 11-week old male diabetic db/db mice. (D) Unaltered SNP relaxation in aorta from 11-week old diabetic db/db male mice (n=12) compared to normoglycaemic db/db+ (n=12) male mice. (E) Unaltered relaxations induced by the NO donor sodium nitroprusside (SNP) in aorta from 11- and 16-week db/db+ male mice. (F) Impaired SNP relaxations in aorta from 16-week male db/db mice compared to 11-week old male diabetic db/db mice. The results are means±SEM. *P<0.05, 16-week old db/db versus 11-week old db/db mice.

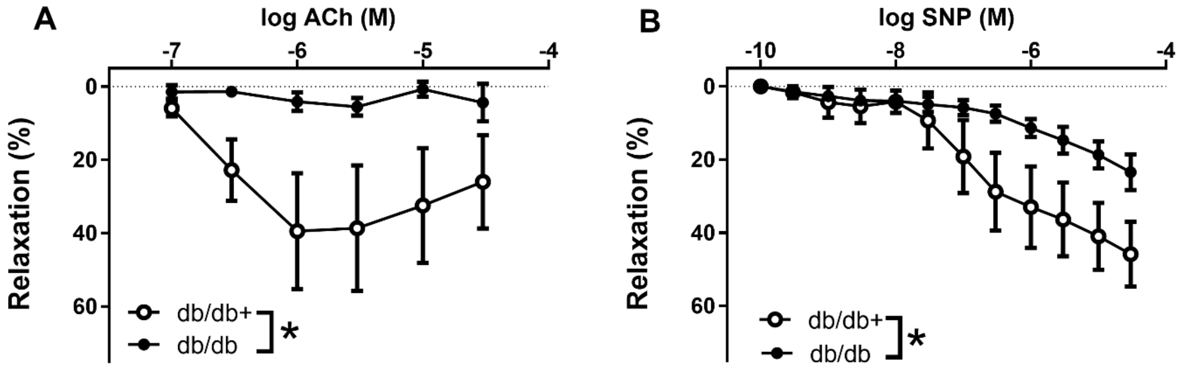

Supplementary Figure S3. Impaired acetylcholine and sodium nitroprusside relaxations in coronary arteries contracted by the thromboxane analog, U46619. Average coronary artery relaxations induced by (A) acetylcholine (ACh) in left anterior descending (LAD) coronary arteries from male db/db+ (n=5) and diabetic db/db mice (n=5). (B) Relaxations induced by sodium nitroprusside (SNP), an NO-releasing drug in LAD from db/db+ control (n=6) and male diabetic db/db animals (n=6). The results are means±SEM. *P<0.05 db/db versus db/db+ control mice.

2.2. ECG measurements

The duration of the PR-interval, QT-interval and the corrected QT-interval on the ECG were significantly prolonged in diabetic animals (supplementary Figure S4).

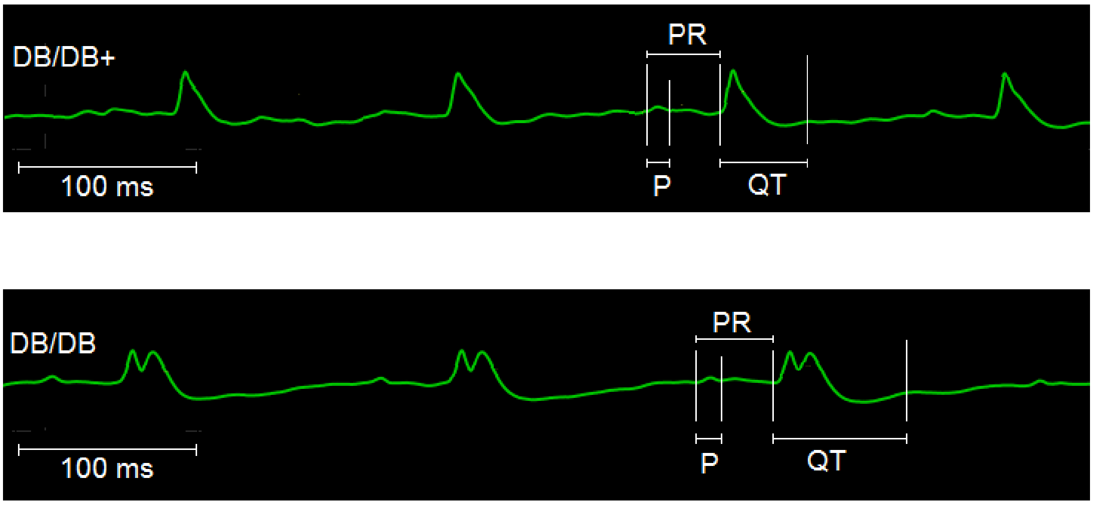

Supplementary Figure S4. Representative electrocardiogram from a diabetic db/db mouse and a control db/db+ mouse. Note the M-shaped QRS complex.

2.3. Transthoracic echocardiography

Echocardiography of aorta showed decreased mean flow and maximal flow velocity in diabetic db/db mice (Supplementary Figure S5).

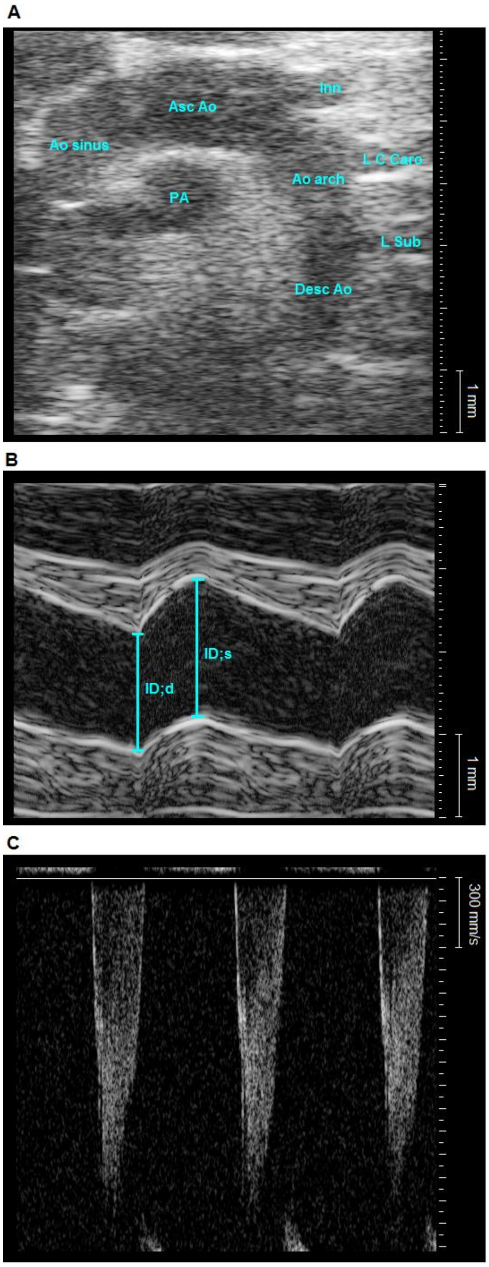

Supplementary Figure S5. Representative echocardiographic images of aorta in a db/db mouse. Representative images of the aortic arch (A, B-mode), the ascending aorta (B, M-mode) and flow through the aortic arch (C, pulse wave Doppler mode). Ao: aorta, Asc: ascending, d: diastole, Desc: descending, ID: inner diameter, Inn: innominate artery, L. C. Caro: left common carotid artery, L Sub: left subclavian artery, PA: right pulmonary artery.
